# Pulmonary endometriosis presenting as multiple nodules and pseudocavities

**DOI:** 10.1002/rcr2.1402

**Published:** 2024-06-19

**Authors:** Zixuan Liu, Xiaoli Deng, Yanhong Du, Danxiong Sun

**Affiliations:** ^1^ Department of Orthopedics Beijing Jishuitan Hospital Guizhou Hospital Guiyang China; ^2^ Department of Respiratory and Critical Care Medicine The First People's Hospital of Yunnan Province Kunming China

**Keywords:** cyclic clinical symptoms, hemoptysis, pseudocavity, pulmonary endometriosis, pulmonary nodule

## Abstract

Pulmonary endometriosis is a rare disease of uncertain pathogenesis which generally presents with the cyclic clinical symptoms and catamenial changes noticed on computer tomography during menstruation. We report a case of a 33‐year‐old woman with recurrent hemoptysis for 1 year. The patient did not exhibit a temporal relationship between her periods and the onset of hemoptysis. A chest computed tomography scan showed multiple pseudocavities in the lower lobe of the right lung and multiple nodules in both lower lobes of the lungs. The right lower lobe wedge resection was performed. Postoperative pathological examination showed pulmonary endometriosis which is a rare cause of hemoptysis.

## Graphical abstract

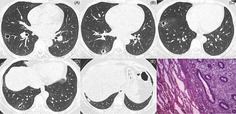

## INTRODUCTION

Typical pulmonary endometriosis manifests as catamenial respiratory symptoms and radiographic findings during on the menstrual cycle. The most common radiological manifestations are pneumothorax and ill‐defined or well‐defined opacities, followed by pulmonary nodules. Lung cavities is the least common entity found in pulmonary endometriosis. In our case, the patient's hemoptysis was not synchronized with the menstrual cycle, and the chest computed tomography (CT) showed multiple nodules and pseudocavities.

## CASE REPORT

A 33‐year‐old woman with no past medical history sought treatment at the hospital with recurrent hemoptysis for 1 year. She experienced hemosputum which was not related to the menstrual cycle. She did not report fever, weakness, anorexia, night sweats, weight loss, expectoration, dyspnea, or chest pain. About 2 months before admission, she coughed up bright red blood. The volume of hemoptysis was about 50 mL. She was diagnosed with bacterial pneumonia and treated with antibiotics and hemostatic drugs with resolution of her symptom in a local hospital. She was admitted to the hospital for further evaluation. She is married, having a healthy daughter. The patient has no history of dysmenorrhrea or other gynaecological diseases. No history of pelvic surgery.

**FIGURE 1:**
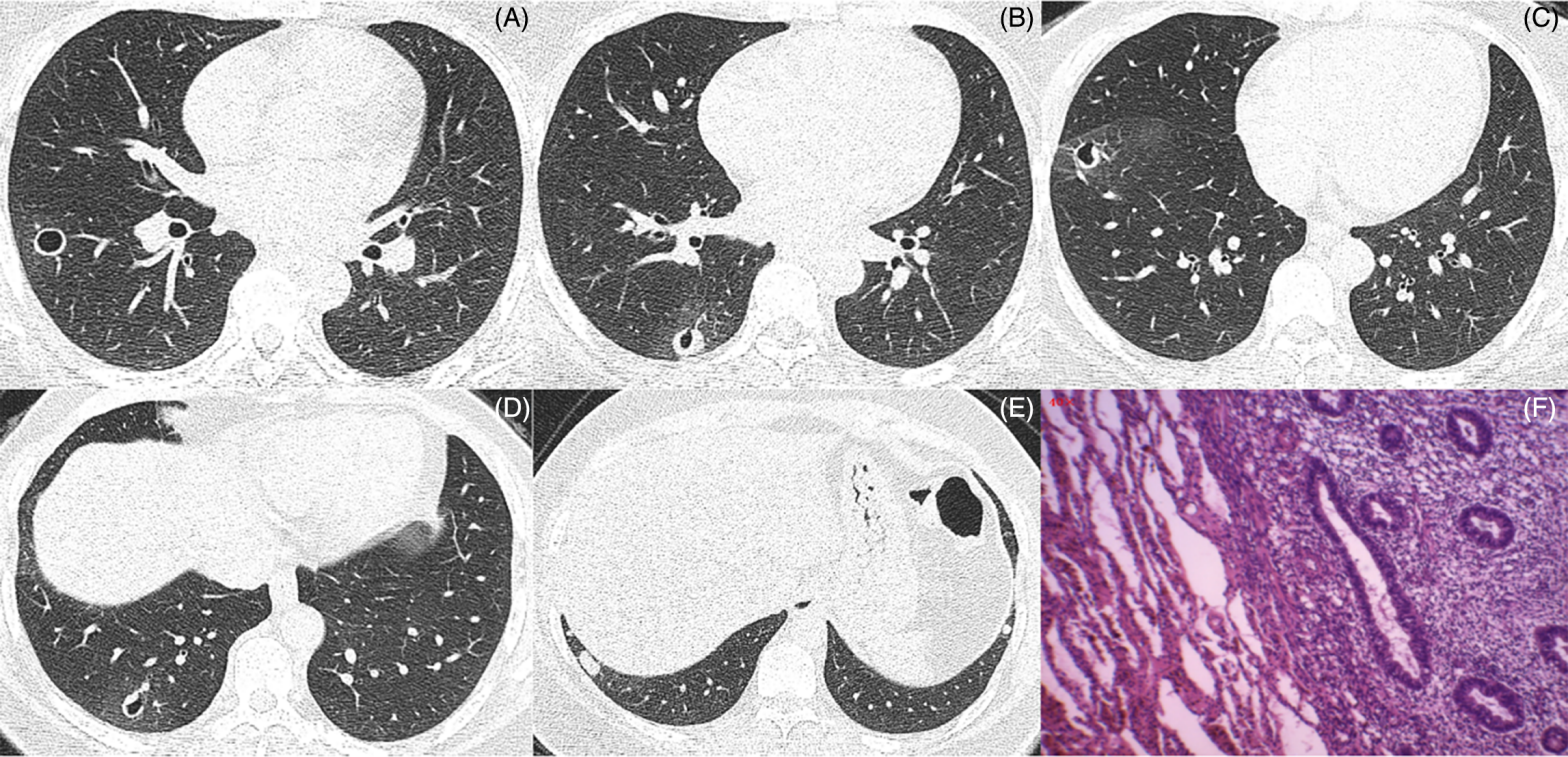
The chest computed tomography scan showed a circular cavity with a smooth inner wall in the lower lobe of the right lung (A). Multiple pseudocavities in the lower lobe of the right lung (B–D). Multiple nodules in both lower lobes of the lungs (E). (H&E, ×40) Microscopic examination of the lung nodule revealed lung parenchyma (left side) and ectopic endometrial glands (right side) (F).

The physical examination findings were unremarkable. Routine blood test was normal except for an mildly elevated percentage of monocytes of 10.4%. Urine routine: protein (+), the rest is normal. Liver function test results were unremarkable except for an mildly elevated globulin level of 35 g/L. Fibrinogen was 4.16 g/L and other coagulation function related indicators were normal. The renal function panel was within normal range. Antineutrophil cytoplasmic antibody‐associated vasculitis was negative. The antinuclear antibodies titre was 1:100, and the rest of the autoimmune serological tests were negative. The whole blood IFN‐γ release assay was negative. Spirometric testing and echocardiography were normal. The chest CT scan showed a circular cavity with a smooth inner wall and multiple pseudocavities in the lower lobe of the right lung and multiple nodules in both lower lobes of the lungs (Figure 1A–E). The cavity and pseudocavities were surrounded by ground‐glass opacities. Tracheobronchial tree inspection results by flexible video bronchoscopy were normal. The smear and culture of bronchoalveolar lavage fluid for fungi, acid‐fast bacilli, and other bacteria were all negative.

Because there was no conclusive answer for the cause of the pulmonary lesion, a right‐sided video‐assisted thoracoscopy was performed with lung biopsy. Histopathologic examination of the wedge resected specimens showed typical pulmonary endometriosis (Figure 1F). The patient's postoperative course was uneventful and was discharged without any symptoms. The patient refused treatment because she felt that recurrent hemosputum had no impact on her life. Following 4 years of follow‐up, the patient maintains an active life, although she still has recurrent hemosputum.

## DISCUSSION

Endometriosis is defined as an extrauterine growth of endometrial tissue. Pulmonary endometriosis is a rare form of thoracic endometriosis. All the theories that have been proposed, including retrograde menstruation, microembolization theory, coelomic metaplasia and stem cell theory, fail to clearly state the mechanism of pulmonary endometriosis.^1^

Endometriosis within the lung parenchyma produces a range of clinical manifestations including catamenial pneumothorax, hemothorax, hemoptysis, chronic cough, and recurrent episodes of low‐grade fever. Additionally, as in our case, not all patients exhibit a temporal relationship between the periods and symptoms. It is important to note that pulmonary endometriosis also can be asymptomatic.^2^ Pulmonary endometriosis has no specific x‐ray and CT manifestations, and the most common radiological manifestations are pneumothorax and opacities. It rarely can appear in nodule, cavity or bullous formations.^3^ In a few cases, it masqueraded as central‐type lung cancer.^4^ Kiyan et al.^5^ reported an unusual radiographic finding in pulmonary parenchymal endometriosis which manifested as bilateral multiple ring‐shaped lesions. In the present case, CT of the chest showed multiple pseudocavities. To our knowledge, this radiological finding has not been reported before. We speculate that its formation mechanism may be the circular invasion of endometrial tissue into lung tissue.

No definitive guidelines for the treatment of pulmonary endometriosis have yet been established. In general, drug therapy is the first‐line treatment. After failure of drug treatment, operative treatment is the choice of most patients. A few studies have shown that percutaneous cryoablation may be an ideal and effective treatment option for patients with multiple endometriosis and if the lung lesions are superficial and limited, photodynamic therapy can a treatment option for patients who develop recurrent hemoptysis after drug withdrawal.^6^, ^7^ In the present case, the patient refused treatment. Following 4 years of follow‐up, she maintains an active life, although she still has recurrent hemosputum. Given the risk of failure and adverse reactions in drug therapy, the patient's choice may be appropriate.

Unless there is a temporal association between patients' pulmonary symptoms and menstruation and the physicians have a high clinical suspicion, the diagnosis of pulmonary endometriosis often goes unrecognized. The differential diagnosis of pseudocavities should include pulmonary endometriosis. A definitive diagnosis requires pathological examination.

## AUTHOR CONTRIBUTIONS

Data acquisition, Zixuan Liu. All authors contributed to the writing and revision of this manuscript.

## CONFLICT OF INTEREST STATEMENT

None declared.

## ETHICS STATEMENT

The authors declare that appropriate written informed consent was obtained for publication of this manuscript and the accompanying images.

## Data Availability

The data that support the findings of this study are available on request from the corresponding author. The data are not publicly available due to privacy or ethical restrictions.
